# Influenza pandemic intervention planning using *InfluSim*: pharmaceutical and non- pharmaceutical interventions

**DOI:** 10.1186/1471-2334-7-76

**Published:** 2007-07-13

**Authors:** Hans P Duerr, Stefan O Brockmann, Isolde Piechotowski, Markus Schwehm, Martin Eichner

**Affiliations:** 1 Department of Medical Biometry, University of Tübingen, Germany; 2 Baden-Württemberg State Health Office, District Government Stuttgart, Germany

## Abstract

**Background:**

Influenza pandemic preparedness plans are currently developed and refined on national and international levels. Much attention has been given to the administration of antiviral drugs, but contact reduction can also be an effective part of mitigation strategies and has the advantage to be not limited *per se*. The effectiveness of these interventions depends on various factors which must be explored by sensitivity analyses, based on mathematical models.

**Methods:**

We use the freely available planning tool *InfluSim *to investigate how pharmaceutical and non-pharmaceutical interventions can mitigate an influenza pandemic. In particular, we examine how intervention schedules, restricted stockpiles and contact reduction (social distancing measures and isolation of cases) determine the course of a pandemic wave and the success of interventions.

**Results:**

A timely application of antiviral drugs combined with a quick implementation of contact reduction measures is required to substantially protract the peak of the epidemic and reduce its height. Delays in the initiation of antiviral treatment (e.g. because of parsimonious use of a limited stockpile) result in much more pessimistic outcomes and can even lead to the paradoxical effect that the stockpile is depleted earlier compared to early distribution of antiviral drugs.

**Conclusion:**

Pharmaceutical and non-pharmaceutical measures should not be used exclusively. The protraction of the pandemic wave is essential to win time while waiting for vaccine development and production. However, it is the height of the peak of an epidemic which can easily overtax general practitioners, hospitals or even whole public health systems, causing bottlenecks in basic and emergency medical care.

## Background

The recent spread of highly pathogenic avian influenza from Asia to Europe and the transmission to humans has intensified concerns over the emergence of a novel strain of influenza with pandemic potential. While still being in an inter-pandemic stage, nations plan for pandemic contingency following recommendations of the WHO [1,2]. National influenza preparedness plans are constantly being refined, aiming to mitigate the effects of pandemic influenza on a national, regional and local level. Even in the absence of a pandemic strain, seasonal influenza causes substantial morbidity and mortality [3]. Seasonal outbreaks put pressure on general practitioners and strain hospital resources, leading to bottlenecks in outpatient treatment and hospital admission capacities.

Various intervention strategies reduce the impact of influenza on individuals and public health systems. In inter-pandemic phases, vaccination is the most important tool to reduce morbidity and mortality, but a potent vaccine will probably not be generally available in the initial phase of a pandemic [4]. Other control strategies like pharmaceutical (antiviral) [5,6] and non-pharmaceutical interventions (reduction of contact rates) [7,8] will have to be implemented.

The use of antiviral drugs during a pandemic seems to be the treatment of choice at present [9-12], but not all countries can afford stockpiling enough drugs. Furthermore, concerns about the over-reliance of a "pharmaceutical solution" have been expressed [13]. An epidemic can also be mitigated by reducing contact rates in the general population and by decreasing the infectivity of cases [9]. Such reductions can be achieved by measures like quarantine and case isolation [14], closing day care centres and schools, cancelling mass gathering events, voluntary self isolation and general behavioural changes in public and increasing social distance [8].

The effectiveness of such interventions depends on various factors which must be prospectively explored by sensitivity analyses, based on mathematical models. Here, we use the freely available Java applet *InfluSim *[15] to investigate how effectively pharmaceutical and non-pharmaceutical interventions contribute to mitigate an influenza pandemic while vaccines are not available. In particular, we examine how intervention delays determine the course of a pandemic and constrict the success of interventions.

## Methods

*InfluSim *is a deterministic compartment model based on a system of over thousand differential equations which extend the classic SEIR model by clinical and demographic parameters relevant for pandemic preparedness planning. Details of the simulation and a discussion of the standard parameter values have been described previously [15]; a summarizing description of the model is provided in the Appendix. The program and its source code are publicly available [16] to offer transparency and reproducibility. The simulation produces time courses and cumulative numbers of influenza cases, outpatient visits, applied antiviral treatment doses (neuraminidase inhibitors), hospitalizations, deaths and work days lost due to sickness, all of which may be associated with financial loss. The analyses presented here are based on *InfluSim *2.0, using demographic and public health parameters which represent the situation in Germany in 2006. Interventions include antiviral treatment, isolation of patients, social distancing measures and the closing of day care centres and schools as well as cancelling mass gathering events.

Using the standard set of InfluSim parameters (freely accessible from [15]), about one third of all infected individuals is expected to become severely ill and to seek medical help. Patients seeking medical help will be referred to as "outpatients" throughout this paper. An exponential distribution is used to model the delay between onset of symptoms and seeking medical help; on average, patients visit a doctor after 24 hours. If a patient seeks medical help within 48 hours after onset of symptoms, he or she is given antiviral treatment unless the stockpile of antivirals is exhausted. Antiviral treatment reduces the duration and degree of infectivity of the case and the number of hospitalizations (Table 1) [17]. For more detailed descriptions see [15] or the Appendix.

**Table 1:** Antiviral treatment schedule and effects

Parameter	Value	Source
Average time for seeking medical help after symptom onset	*D*_D _= 24 h	assumed
therapeutic window (after onset of symptoms)	*D*_T _= 48 h	Fachinformation Roche
fraction eligible to receive treatment		assumed
• severe cases who can stay at home	*f*_V _= 100%	
• extremely severe cases who need hospitalization	*f*_X _= 100%	
treatment reduces the duration of the infectious period	*f*_D _= 25%	Fachinformation Roche
treatment reduces infectiousness by	*f*_I _= 80%	Longini (2004)
treatment reduces hospitalizations by	*f*_H _= 50%	Kaiser (2003)

Non-pharmaceutical interventions examined in this paper are contact reduction measures and the isolation of cases. The latter effectively leads to reduced contact rates between individuals, too. In the scenarios presented below, we assume that everybody in the population avoids a given percentage of contacts (e.g. by improved hygiene, wearing masks, or behavioural changes) and that sick patients are isolated which reduces the contact rates of moderately sick, severely sick (but non-hospitalized) and hospitalized cases by 10%, 20% and 30%, respectively. Further interventions which comprise the closing of day care centres and schools, and the cancelling of mass gathering events will be examined in detail in a separate paper.

## Results

**Figure 1:**
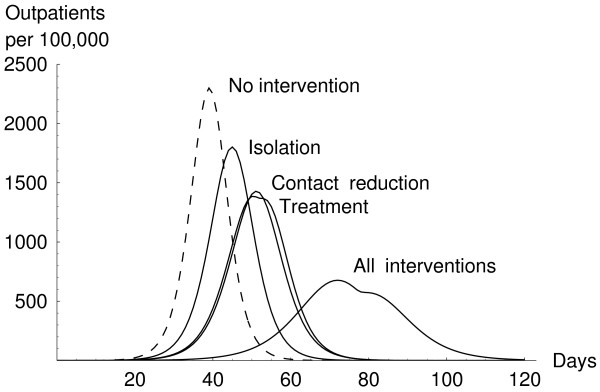
**Comparison of different intervention schemes**. Number of outpatients expected during a pandemic wave in a population of 100,000 citizens. Parameter values are based on the *InfluSim *standard configuration [15] with *R*_0 _= 2.5, except those listed at the end of this legend and indicated by superscripts^1^. The dashed line represents an epidemic without intervention^2^. For the following four scenarios, interventions are initiated when infection is introduced (day 0). Isolation: moderately sick, severely sick and hospitalized cases are isolated^3^. Treatment: antivirals are available for 10% of the population and all severe and extremely sick cases receive antiviral treatment^4^. Under this intervention scheme, antivirals are used up on day 50. Contact reduction: involves isolation^3 ^of cases and social distancing^5^. All interventions: combination of all three interventions^6^; under this intervention scheme, antivirals are used up on day 76, leading to a plateau in the epidemic curve. ^1^:Parameter modifications are given in the following and terms in italics refer to terms in the *InfluSim *user interface. *InfluSim *output: *N*_i _= cumulative proportion of the population infected, and *N*_o _= cumulative proportion of outpatients in the population. ^2^: yielding *N*_i _= 87%, *N*_o _= 29% ^3^: *Moderately sick cases*: 10%, *Severe cases (home)*: 20%, *Severe cases (hospital)*: 30%, yielding *N*_i _= 81%, *N*_o _= 27%. ^4^: *Antivirals availability*: 10%, *Treatment fraction*: 100% for both, *Treatment of severe cases *and *Treatment of extremely sick cases*, yielding *N*_i _= 82%, *N*_o _= 27%. ^5^: *General reduction of contacts*: *Contact reduction by *10%. Combined with isolation of cases, this intervention scheme yields *N*_i _= 75%, *N*_o _= 25%. ^6^: yielding *N*_i _= 66%, *N*_o _= 22%.

Assuming a basic reproduction number of *R*_0 _= 2.5 and using the standard parameter set of *InfluSim *[15], an epidemic in a population of 100,000 individuals reaches the peak about 40 days after introduction of the infection and is practically over three weeks thereafter if no interventions are performed (Figure 1). During the whole epidemic, 87% of the population become infected, 29% seek medical help, 0.7% are hospitalized and 0.2% die. Figure 1 shows how pharmaceutical and non-pharmaceutical interventions can mitigate this scenario. Contact reduction by isolation of cases alone (see Appendix), protracts the peak of the epidemic by about one week. Distribution of antivirals or additional contact reduction measures delay the epidemic by approximately 10 days and are hardly sufficient to provide a substantial delay. A combination of antiviral treatment, isolation of cases and social distancing in the general population seems to be necessary to delay the epidemic in the order of weeks. This example furthermore shows that an efficient mitigation of the epidemic is not necessarily associated with a significant reduction in the number of infections. For information on the proportions of infected people and outpatients see the legends to the Figures.

### Intervention with antivirals

**Figure 2:**
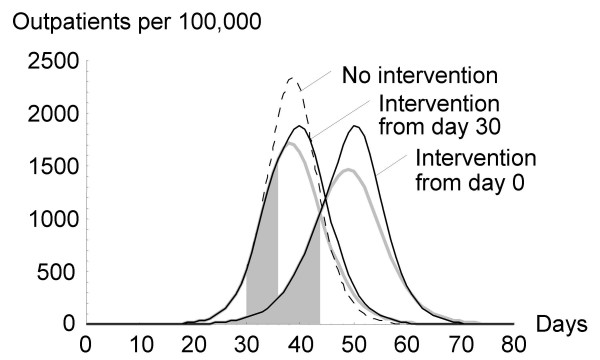
**Onset and sustainability of antiviral intervention**. Number of outpatients expected during a pandemic wave, varied by day of onset when antivirals come into operation. Parameter values are based on the *InfluSim *standard configuration [15] with *R*_0 _= 2.5, except those listed at the end of this legend and indicated by superscripts^1^. The dashed curve shows the epidemic without intervention^2^. Antivirals are available for 5% of the population^3 ^(black lines), compared to scenarios of full coverage^4 ^(grey lines). The shaded areas under the curves represent the amounts of antivirals distributed and are identical for both scenarios. They are shown between onset of intervention and exhaustion. If antivirals are available at the beginning of the epidemic ("Intervention from day 0") they last for 45 days^5^. Antivirals last only for a shorter period, if coming into operation in later phases of the epidemic ("Intervention from day 30")^6^. ^1^:Parameter modifications are given in the following and terms in italics refer to terms in the *InfluSim *user interface. *InfluSim *output: *N*_i _= cumulative proportion of the population infected, and *N*_o _= cumulative proportion of outpatients in the population. ^2^: Yielding *N*_i _= 87%, *N*_o _= 29%. ^3^: *Antiviral availability*: 5%, *Treatment fraction*: 100% for both, *Treatment of severe cases *and *Treatment of extremely sick cases*, yielding *N*_i _= 84%, *N*_o _= 28% for scenarios, "day 0" and "day 30". ^4^: *Antiviral availability*: 100%, *Treatment fraction*: 100% for both, *Treatment of severe cases *and *Treatment of extremely sick cases*, yielding *N*_i _= 72%, *N*_o _= 24% for "day 0" and *N*_i _= 74%, *N*_o _= 25% for "day 30". ^5^: *Antiviral availability*: 5%, *Treatment fraction*: 100%, *Range of days*: 0–80 for both, *Treatment of severe cases *and *Treatment of extremely sick cases*. ^6^: *Antiviral availability*: 5%, *Treatment fraction*: 100%, *Range of days*: 30–80 for both, *Treatment of severe cases *and *Treatment of extremely sick cases*.

The mitigating effect of antivirals strongly depends on the onset of their distribution (Figure 2). Antivirals can delay the epidemic if distributed very early while few cases exist in the population. Late distribution of antivirals (e.g. starting on day 30) leads to the paradoxical effect that the stockpile is exhausted even quicker compared to early distribution (shaded areas und the curves in Figure 2). Additionally, the mitigating effect of the intervention drastically diminishes and benefits are restricted to lowering the peak of the epidemic. Unrestricted availability of drugs (grey curves in Figure 2) still leads to an epidemic because (i) asymptomatic and moderately sick cases are not eligible for treatment, (ii) patients visit a doctor on average 24 hours after onset of symptoms while already being highly infectious and (iii) antivirals cannot fully prevent infectivity.

**Figure 3:**
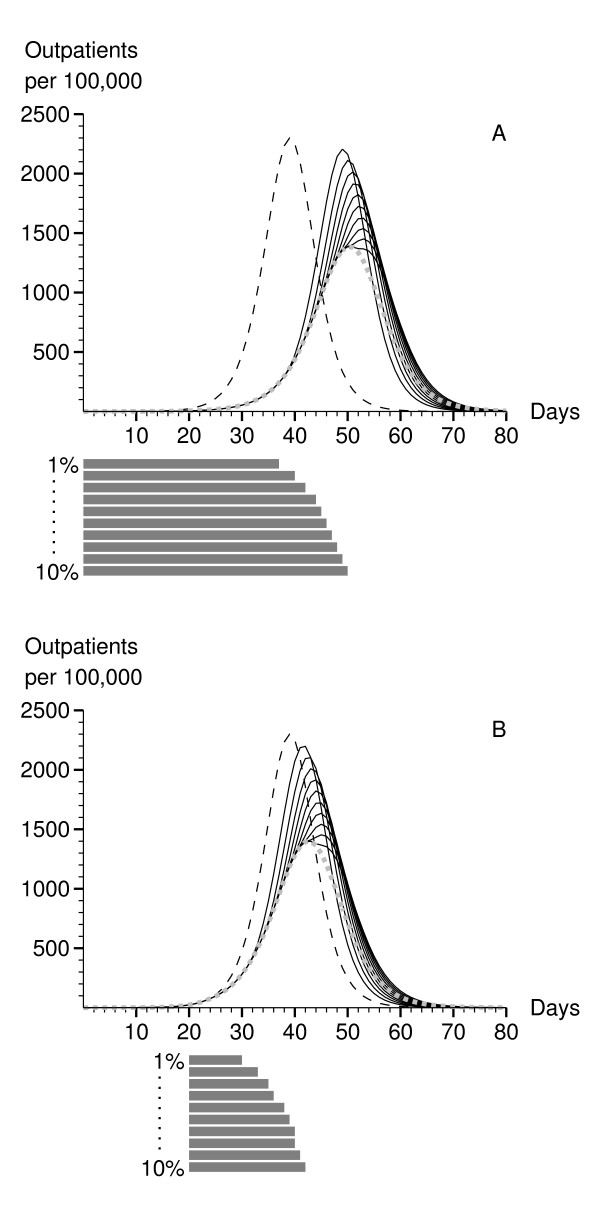
**Intervention with limited amounts of antivirals**. Number of outpatients expected during a pandemic wave, varied by the availability of antivirals. Parameter values are based on the *InfluSim *standard configuration [15] with *R*_0 _= 2.5, except those listed at the end of this legend and indicated by superscripts^1^. *Antiviral availability *ranges from 0% (no antivirals available, dashed curves^2^) to 10% (antivirals available for 10% of the population^3^) in steps of 1% (from left to right). The dashed curve shows the epidemic without intervention. Grey dotted lines represent the scenario where antivirals are available for the whole population^4^. Bars at the bottom of each graph indicate the period when antiviral treatment begins (model input) until stockpiles are used up (model output). **A**: Antivirals are available from day 0 ^5^. **B**: Antivirals become available after three weeks^6^. The epidemic curves depart from the grey dotted line when antivirals are exhausted.^1^:Parameter modifications are given in the following and terms in italics refer to terms in the *InfluSim *user interface. *InfluSim *output: *N*_i _= cumulative proportion of the population infected, and *N*_o _= cumulative proportion of outpatients in the population. ^2^: *Antiviral availability*: 0%, yielding *N*_i _= 87%, *N*_o _= 29% for both, A and B. ^3^: *Antiviral availability*: 10%, yielding *N*_i _= 82%, *N*_o _= 27% for both, A and B. ^4^: *Antiviral availability*: 100%, yielding *N*_i _= 72%, *N*_o _= 24% for both, A and B. ^5^: *Range of days*: 0–80. ^6^: *Range of days*: 21–80.

Figure 3 extends these considerations by showing epidemic curves where all clinically ill patients are treated with antiviral drugs until the stockpile is exhausted. The mitigating effect of antiviral distribution is weakly influenced by the amounts of available antivirals, but is strongly determined by the onset of administration. The model suggests that even a small stockpile of antivirals can protract the peak of the epidemic if distributed very early while few cases exist in the population (Figure 3A). In contrast, the mitigating effect becomes negligible, if antivirals are distributed with delay (Figure 3B). Independent of the delay in the distribution of antivirals, their quantitative availability affects only the height of the peak of the epidemic, but hardly the mitigation of the epidemic (Figure 3A, B). For considerations into the final size of the epidemic see below. In summary, delaying the epidemic depends on early action, whereby lowering the peak depends on the quantitative availability of antivirals.

### Intervention through contact reduction

**Figure 4:**
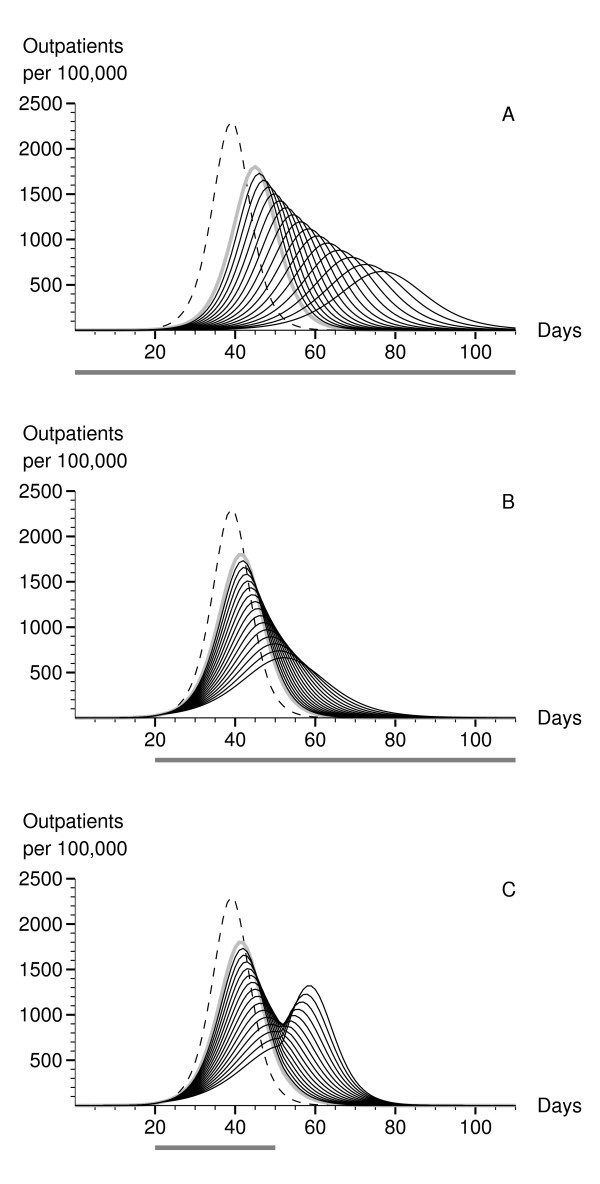
**Effects of contact reduction measures**. Number of outpatients expected during a pandemic wave if contact reduction measures are implemented additionally to the isolation of cases. Parameter values are based on the *InfluSim *standard configuration [15] with *R*_0 _= 2.5, except those listed at the end of this legend and indicated by superscripts^1^. The dashed curve shows the epidemic without intervention. Contact reduction involves social distancing^2 ^and isolation of cases^3^. The curves show the effects caused by social distancing, where contacts are reduced by 0%^4 ^(grey curve) up to 30%^5 ^in steps of 2%^6 ^(black curves, from left to right). Bars at the bottom of each graph illustrate the periods of contact reduction, which are in **A**: full, from day 0 to end, in **B**: delayed, from day 20 to the end, and in **C**: temporarily, from day 20 to day 50.^1^:Parameter modifications are given in the following and terms in italics refer to terms in the *InfluSim *user interface. *InfluSim *output: *N*_i _= cumulative proportion of the population infected, and *N*_o _= cumulative proportion of outpatients in the population. ^2^: *Contact reduction: *ranging from 0–30% in steps of 2%. *Range of days*: varied between A, B, and C, see legend or grey bar at the bottom of each graph. ^3^: *Isolation*: *Moderately sick cases*: 10%, *Severe cases (home)*: 20%, *Severe cases (hospital)*: 30%. *Range of days*: varied between A, B, and C, see legend or grey bar at the bottom of each graph. ^4^: Intervention effect is based on *Isolation *alone, yielding *N*_i _= 81%, *N*_o _= 27% in A, B and C. ^5^: Yielding in A: *N*_i _= 56%, *N*_o _= 19%, and B: *N*_i _= 57%, *N*_o _= 19%, and C: *N*_i _= 82%, *N*_o _= 27%. ^6^: E.g. for a *Contact reduction *of 20%, we obtain in A: *N*_i _= 68%, *N*_o _= 22%, in B: *N*_i _= 67%, *N*_o _= 22%, and in C: *N*_i _= 79%, *N*_o _= 26%.

Contact reduction measures, comprising social distancing and the isolation of cases, can be an effective part of mitigation strategies; they have the advantage over antiviral treatment to be not limited *per se*, i.e. they can be continued for a sufficiently long period of time. Figure 4 examines the effect of isolation of cases and social distancing measures (see figure caption for details) in the absence of antiviral treatment. The peak of the epidemic is protracted by about 1 day for every percent of contact reduction if this intervention starts immediately after the introduction of the infection. Thus, a peak shift is not only possible by early action, but also by the degree of contact reduction. If contact reduction is initiated later, the peak shift diminishes, but the proportionality remains. For example, if the intervention starts three weeks after the introduction of infection, the peak of the epidemic is only mitigated by about half a day per 1% contact reduction (Figure 4B). Premature cessation of contact reduction measures restores the infection rates to the pre-intervention values which fuels the epidemic. It can lead to a delayed course and a higher total number of infections, involving a plateau or even a second peak of the epidemic (Figure 4C).

### Combined intervention scheme

**Figure 5:**
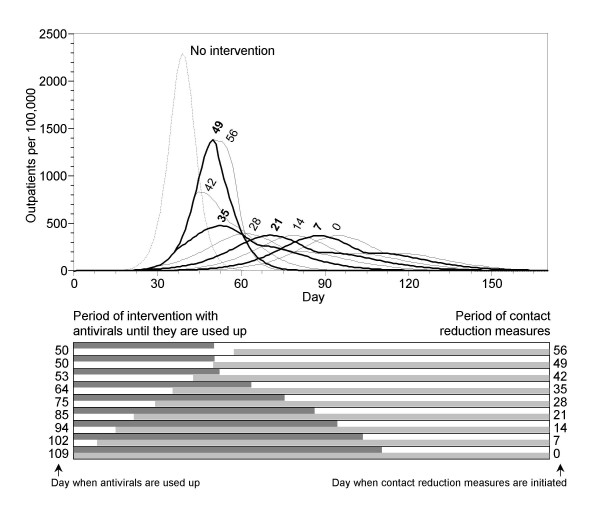
**Combined intervention scheme**. Number of outpatients expected during an influenza pandemic if antiviral distribution and contact reduction measures are implemented additionally to the isolation of cases. Parameter values are based on the *InfluSim *standard configuration [15] with *R*_0 _= 2.5, except those listed at the end of this legend and indicated by superscripts^1^. The Figure shows the epidemic curves, varied by the "Day when contact reduction measures are initiated" (as indicated by the number next to the peak and at the right hand side of the bar chart). Antivirals are available for 10% of the population and are distributed from day zero^2^. Contact reduction measures involve isolation of cases^3 ^and social distancing^4^. Bars at the bottom of the graph illustrate begin and end antiviral intervention (dark bars) and contact reduction measures (light bars), respectively. The "Day when contact reduction measures are initiated" is model input, whereas the "Day when antivirals are used up" is model output. The epidemic without intervention is shown as a dashed curve. The curves for "7", "21", "35" and "49"^5 ^are plotted in bold for purposes of visualization.^1^:Parameter modifications are given in the following and terms in italics refer to terms in the *InfluSim *user interface. *InfluSim *output: *N*_i _= cumulative proportion of the population infected, and *N*_o _= cumulative proportion of outpatients in the population. ^2^: *Antiviral availability*: 10%. *Treatment fraction*: 100%, for both, *Treatment of severe cases *and *Treatment of extremely sick cases*. ^3^: *Moderately sick cases*: 10%, *Severe cases (home)*: 20%, *Severe cases (hospital)*: 30%. *Range of days*: see bar chart at the bottom of the graph. For "day 0", *N*_i _= 53%, *N*_o _= 18%. ^4^: *Contact reduction by*: 20%. *Range of days*: see bar chart at the bottom of the graph. ^5^: Yielding for scenarios up to "day 28": *N*_i _= 53%, *N*_o _= 18%, for "day 35": *N*_i _= 55%, *N*_o _= 18%, for "day 42": *N*_i _= 60%, *N*_o _= 20%, for "day49": *N*_i _= 69%, *N*_o _= 23%.

The preceding examples with interventions based on antivirals or contact reduction alone yielded peak delays only in the order of weeks, whereas months may be required for vaccine development and production, demanding for a combined intervention scheme (Figure 5). We examine an optimistic scenario where antivirals are distributed immediately after the infection is introduced (dark bars in Figure 5), while varying the onset of social distancing measures. The antiviral stockpile lasts longer if social distancing measures are initiated earlier (pale bars in Figure 5). Immediate initiation of contact reduction can protract the epidemic by months, whereas a delayed initiation leads to a plateau in the epidemic curve at a time when antivirals are used up.

### Cumulative number of infections and outpatients

Without interventions, *N*_i _= 87% of the population become infected during the course of the epidemic and the cumulative number of outpatients reaches *N*_o _= 29%, reflecting the assumption that approximately one third of infected individuals becomes sufficiently sick to seek medical help. These outcomes remain surprisingly stable even for interventions assuming optimistic resources (cf. footnotes to Figures 1, 2, 3, 4, 5). For instance, immediate and unlimited availability of antivirals reduces these fractions only to *N*_i _= 72% and *N*_o _= 24% (Figure 2). This minor effect has three reasons: only about one third of cases seeks medical help and will receive antiviral treatment, many infections are passed on before cases seek medical help and antiviral treatment does not fully prevent further transmission. These disadvantages do not apply to contact reduction measures. For instance, a reduction of 20% of contacts reduces these fractions to *N*_i _= 68% and *N*_o _= 22% (Figures 4A, B). A combination of antiviral treatment and contact reduction can further reduce these values to *N*_i _= 53% and *N*_o _= 18% (Figure 5).

### Uncertainty in the parameter values

**Figure 6:**
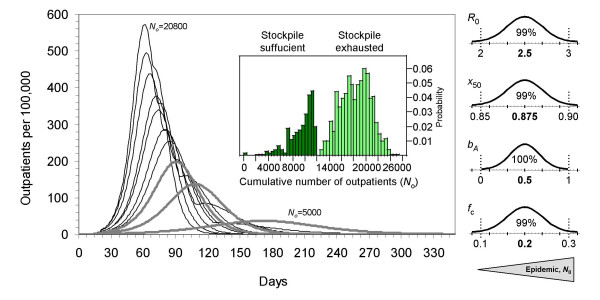
**Sensitivity analysis considering uncertainty of parameter values**. Variability in epidemic curves (large plot) and the distribution of the cumulative number of outpatients (inset), originating from the uncertainty in four parameters (right panel). Parameter values are based on the *InfluSim *standard configuration [15] with *R*_0 _= 2.5, except those listed at the end of this legend and indicated by superscripts^1^. The sensitivity analysis extends the scenario shown in Figure 5, where antivirals are available for 10% of the population and are distributed from day zero^2^, and where contact reduction measures^3^, including the isolation of cases^4^, are initiated three weeks after the introduction of infection (scenario "day 21"). **Right panel**: parameter values for each realization are sampled independently from normal distributions as shown (means given in bold, 99% of the values lie within the range specified by dotted lines, except *b*_A _which is truncated). *R*_0_: basic reproduction number, *x*_50_: cumulative infectivity during the first half of the symptomatic period, *b*_A_: relative infectivity of asymptomatic cases, *f*_c_: antiviral treatment reduces infectivity by a factor of 1-*f*_c_. For each parameter, an increase of the value aggravates the epidemic. **Large plot**: from a hundred random realizations, we selected the two most extreme epidemics, and eight epidemics homogeneously placed between them. The epidemic with *N*_0 _= 20800 is caused by parameter values drawn from the left tail of the corresponding distributions, and the epidemic with *N*_0 _= 5000 is caused by parameter values drawn from the right tail of the corresponding distributions (see right panel). The epidemic curves show a plateau or a second wave when antiviral stockpiles are exhausted while the proportion of susceptibles is still large enough to allow for further propagation of infectives (thin curves in black); for optimistic parameter combinations (e.g. small *R*_0_), the available stockpiles last over the whole period of the intervention and the epidemic curve proceeds without a plateau (bold curves in grey). **Inset**: distribution of cumulative number of outpatients obtained from 1,000 random realizations.^1^:Parameter modifications are given in the following and terms in italics refer to terms in the *InfluSim *user interface. ^2^: *Antiviral availability*: 10%. *Treatment fraction*: 100%, for both, *Treatment of severe cases *and *Treatment of extremely sick cases*. ^3^: *Contact reduction by*: 20%. *Range of days*: day 21–360. ^4^: *Moderately sick cases*: 10%, *Severe cases (home)*: 20%, *Severe cases (hospital)*: 30%. *Range of days*: day 21–360.

In the preceding analyses it was assumed that parameter values are precisely known; in a real world scenario, however, uncertainty arises from biological variability, stochastic influences, heterogeneities, etc. We illustrate with a concluding example to which extent simulated epidemics are affected by uncertainty in the parameter values. As shown in Figure 6, epidemics can be highly variable, although only four parameters have been varied within moderate ranges. Varying more parameters would further increase this variability.

For the interventions and parameter variations considered, the cumulative number of outpatients ranges from a few thousand to over twenty thousand (see inset in Figure 6). Among the four parameters, *R*_0 _is the strongest predictor of the number of outpatients (analysis not shown) as it strongly determines how quickly antivirals become exhausted. In two out of 1,000 simulations the randomly chosen parameter combinations involved values for *R*_0 _around 1.8 which led to very minor outbreaks given the intervention scheme. The cumulative number of outpatients escalates when antiviral stockpiles become exhausted while the proportion of susceptibles is still large enough to allow for further propagation of infectives. In this case, the epidemic curve proceeds with a second wave or a plateau.

## Discussion

With pandemic influenza, we have to "expect the unexpected" [18]. Historical reports frequently mention the surprising speed at which a pandemic wave travels through the population [19-21]. Predicting the course of a future pandemic which will be caused by a virus with unknown characteristics is based on substantial uncertainties and we must rely on sensitivity analyses, performed with mathematical models like *InfluSim*.

Because of the short serial interval of influenza, timely action is essential. Different control measures must be regarded as complementary and not as competing. Neither antiviral treatment nor non-pharmaceutical measures should be used exclusively to mitigate a pandemic influenza wave.

### Antivirals

Infectious disease models have suggested that an upcoming influenza epidemic with a low basic reproduction number might be contained at the source through targeted use of antiviral drugs [9,12]. The published scenarios concern WHO phases 4 and 5 (inter-pandemic alert period) and assume that an outbreak starts in a rural area with low population density. It can be expected that the pandemic virus will be introduced into Europe and the US after a local epidemic (i.e. in WHO phase 6). Community-based prophylaxis, however, is of limited use for several reasons. Under a high prevalence of infection in phase 6, a wide distribution requires an enormous number of antiviral courses; with available stockpiles, it will be virtually impossible to locally contain the pandemic with targeted antiviral prophylaxis. Development of resistance, limited production capacities and extremely high costs are further limitations of this strategy, so that population-wide prophylaxis has not been recommended by the WHO for the final phase of the pandemic [1].

The discussion of pandemic influenza preparedness planning has frequently focussed on the amounts of drugs to be stockpiled and to whom and when they should be supplied [22]. Even if the currently stockpiled antiviral drugs will be fully effective against the pandemic strain, their use may not be able to sufficiently prevent the spread of influenza because (i) transmission of the infection may occur before the onset of clinical symptoms (as assumed in the InfluSim model) [23], (ii) asymptomatic and moderately sick cases [6] are usually not treated despite contributing to transmission, and (iii) the occurrence of cases with influenza-like illness caused by other pathogens may lead to an accelerated depletion of the antiviral stockpile. Likewise, moderately sick cases or even healthy people may seek medical help and succeed in receiving antiviral treatment which would further deplete the stockpile. These factors reduce the efficacy of pharmaceutical control measures [24], indicating the demand of extending this strategy by non-pharmaceutical intervention measures.

Especially if antivirals are limited, they should be supplied as early as possible. If their distribution is delayed, cases become so abundant that resources will quickly be exhausted without having much impact on the spread of the disease (Figures 2 and 3). This confirms that the amount of antivirals needed strongly depends on the number of infections that are present when the intervention is initiated [25]. If antiviral drugs are extremely limited, they should be used to preferably treat severe cases that need hospitalization. Although this has practically no effect on the pandemic wave *per se*, it helps to reduce the death toll in the population (results not shown).

### Contact reduction

Rather than relying on a pharmaceutical solution, pandemic preparedness should also involve non-pharmaceutical measures (see above). Early self-isolation and social distancing measures can be highly effective, as shown for the SARS epidemic [26]: after the WHO's global alert and the implementation of massive infection control measures, the effective reproduction numbers in Hong Kong, Vietnam, Singapore and Canada fell below unity. Rigorous social distancing measures in the entire population, however, will tax the social and economic structure and the population may not be willing or able to reduce contacts during the whole course of a pandemic wave.

For Figure 5, we assumed that contact reduction measures (e.g. improved hygiene, wearing masks, or behavioural changes) could add up to reduce contacts by 20%. Studies on the SARS outbreak suggest some preventative effect of wearing masks [27-29], but compliance, availability of masks and their effectiveness against influenza infection remain unknown factors. Stockpiling surgical masks for the population results in exorbitant high numbers and may not be feasible [30] and individual stockpiling may be impossible due to economic limitations, especially in crisis situations. Since the specific effects of such behavioral changes remain uncertain, we modeled their contribution as a general reduction in contact rates.

In contrast to SARS, we will not be able to rely on isolating hospitalized cases when a new influenza pandemic emerges. Using the standard parameter settings of *InfluSim*, we expect only a total of 0.7% of the population to be hospitalized. Even for the worst case scenario of the US Pandemic Preparedness Plan, where this value may be up to ten times larger [31], the wide majority of infected individuals is never hospitalized. With influenza, we have to rely on self-isolation of moderately sick cases and of bed-ridden patients who stay at home. As these cases form the majority of infections and exert the highest force of infection, even a moderate reduction of contacts between them and the general population can substantially change the pandemic wave.

## Conclusion

Time is of the essence when controlling infectious diseases that spread at high speed and thus, interventions are most effective in the beginning when only few people are infected. Only a timely application of antiviral drugs (even with limited supplies) and a quick implementation of contact reduction measures will notably protract the peak of the epidemic and substantially reduce its height in a pandemic influenza wave. Whereby the protraction of the pandemic wave is essential to win time while waiting for vaccine development and production, it is the height of the peak of a pandemic wave which can easily overtax general practitioners as well as hospitals and whole public health systems, and can lead to dangerous bottlenecks in basic and emergency medical care. Vaccinating a small fraction of the population with a pre-pandemic vaccine would have a similar effect on the course of the epidemic as reducing the basic reproduction number by the percentage of immunized individuals (e.g. by 10%).

The sensitivity analyses at the end of the Results section shows that the planning of intervention strategies must not only be based on single parameter values, but must also address their variability. More detailed analyses into this will be presented in a subsequent publication. Mathematical models like *InfluSim *should not only be used to predict a specific outcome, but also to explore best and worst case scenarios.

## Competing interests

The author(s) declare that they have no competing interests.

## Authors' contributions

ME developed the model, MS designed the software, HPD wrote the manuscript and SOB and IP formulated the public health requirements of the software. All authors read and approved the final manuscript.

## Appendix: brief description of the transmission dynamics of InfluSim

For a detailed description of *InfluSim *see Eichner M, Schwehm M, Duerr HP, Brockmann SO. The influenza pandemic preparedness planning tool InfluSim. BMC Infect Dis. 2007 Mar 13;7:17.

General description

*InfluSim *is a deterministic compartment model based on a system of over 1,000 differential equations which extend the classic SEIR model by clinical and demographic parameters relevant for pandemic preparedness planning. It allows for producing time courses and cumulative numbers of influenza cases, outpatient visits, applied antiviral treatment doses, hospitalizations, deaths and work days lost due to sickness, all of which may be associated with economic aspects. The software is programmed in Java and open access [16], it operates platform independent and can be executed on regular desktop computers.

Model description

**Figure 7:**
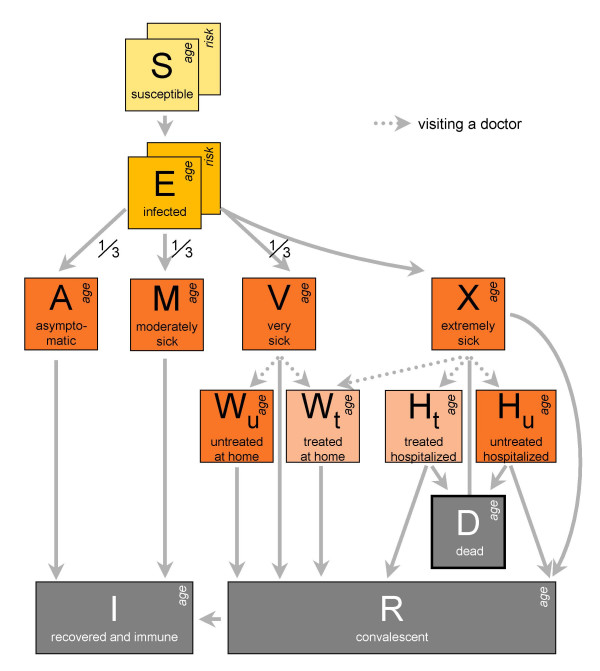
**Model structure of *InfluSim *2.0**. Transitions from each compartment depend on age; transitions from the exposed (E) state into diseased states (A, M, V, X) additionally depend on the risk group which is assigned to susceptible (S) individuals at birth. Other states: W: cases who withdraw at home, H: hospitalized cases, I: recovered and immune individuals, R: individuals in the stage of convalescence, and D: death.

The model structure of *InfluSim *is represented by Figure 7, with descriptions given below.

Natural history of disease

Susceptible individuals (S) are infected at a rate which depends on their age and on the interventions applied at the current time. Infected individuals (E) incubate the infection for a mean duration of 1.9 days. To obtain a realistic distribution of this duration, the incubation period is modelled in 7 stages yielding a gamma distributed incubation period with a coefficient of variation of 37.8%. The last 2 incubation stages are regarded as early infectious period during which patients may already spread the infection. This accounts for an average time of about half a day for the standard set of parameters.

**Figure 8:**
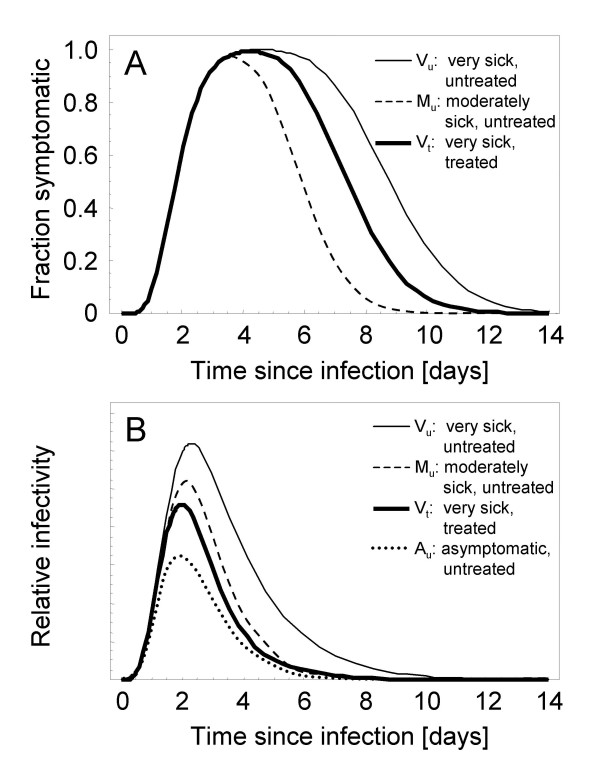
**Time-course of symptoms and infectivity**. Symptoms and infectivity dependent on time, for cases with a severe (V), a moderate (M) or an asymptomatic (A) course of disease (treatment is indicated by subscripts). **A**: Fraction of symptomatic cases among all cases by time since infection. **B**: Relative infectivity by time since infection (given in arbitrary units, as the probability of transmitting the infection also depends on the age-dependent probability of meeting other people as given by the contact matrix).

After passing through the last incubation stage, infected individuals become fully infective and a fraction of them develops clinical symptoms (Figure 8A). The course of disease depends on their age and risk group: one third remains asymptomatic (A), one third shows a moderate course of disease (M, "moderately sick") and the remaining third a severe course of disease (V, "very sick"); a small fraction of the latter third shows an extremely severe course of disease (X, "extremely sick") and needs hospitalization. The rationale for distinguishing extremely sick cases is that only these can die from the disease and need to be hospitalized; in all other aspects, both groups of severe cases are identical. The period of infectivity is gamma distributed and depends on the course of the disease and on the age of the case. To allow for an infectivity which changes over the course of disease, we apply weighting factors which depend on the stage of infectivity. Our standard value results in an infectivity which is highest immediately after onset of symptoms and which declines in a geometric progression over time (Figure 8B).

Severe cases seek medical help on average one day after onset of symptoms, whereby the waiting time until visiting a doctor is exponentially distributed. Very sick and extremely sick patients who visit a doctor may be offered antiviral treatment. Very sick patients are advised to withdraw to their home (W) until the disease is over whereas extremely sick cases are immediately hospitalized (H). Death rates of extremely sick and hospitalized cases are age-dependent. Whereas asymptomatic and moderately sick patients who have passed their duration of infectivity are considered healthy immunes, very sick and extremely sick patients first become convalescent before they resume their ordinary life (gamma distributed with a mean of 5 days and coefficient of variation of 33.3%). Fully recovered patients who have passed their period of convalescence join the group of healthy immunes; working adults will return to work, and children again visit day care centres or schools.

Interventions

**Antiviral treatment**: Severe and extremely severe cases who visit the doctor within at most two days after onset of symptoms are offered antiviral treatment, given that its supply has not yet been exhausted. Antiviral treatment reduces the patients' infectivity by 80 percent, the duration of being diseased by 25%, and the risk of hospitalization by 50 percent. Extremely sick patients, whose hospitalization is prevented by treatment, are sent home and join the group of treated very sick patients.

**Social distancing measures: **Contact rates in the general population can be reduced by increasing "social distance", by closing schools and day care centres, by cancelling mass gathering events, or by behavioural changes.

**Isolation of cases: **Isolation of cases reduces their contact rates. Contacts are not necessarily reduced by 100%, but between 0 and 100%, as specified by the user. Our standard scenario considers reductions of 10%, 20% and 30% for moderately sick cases, very sick cases (at home) and extremely sick cases (hospitalized), respectively.

### Mixing matrix, basic reproduction number and force of infection

For the mixing of the age classes, we employ a "who-acquires-infection-from-whom matrix" (WAIFW matrix) which gives the relative frequency of contacts of infective individuals by age. *InfluSim *assumes bi-directional contacts (e.g. children have the same total number of contacts with adults as adults with children). In order to match the user-specified basic reproduction number *R*_0_, the disease-specific infectivity and the durations of infectivity in this matrix must be incorporated, resulting in the next generation matrix. This matrix is multiplied with a scaling factor chosen such its largest eigenvalue is equal to the chosen value of *R*_0_. The force of infection is given as the product of the number of infective individuals and the corresponding age-dependent contact rates.

Initial values

At the start of the simulation, one infection is introduced into the fully susceptible population. To avoid bias between simulations, the initial infection is distributed over all age and risk classes.

## Pre-publication history

The pre-publication history for this paper can be accessed here:



## References

[B1] WHO WHO global influenza preparedness plan.. http://www.who.int/csr/resources/publications/influenza/GIP_2005_5Eweb.pdf.

[B2] Mounier-Jack S, Coker RJ (2006). How prepared is Europe for pandemic influenza? Analysis of national plans. Lancet.

[B3] Dushoff J, Plotkin JB, Viboud C, Earn DJ, Simonsen L (2006). Mortality due to influenza in the United States--an annualized regression approach using multiple-cause mortality data. Am J Epidemiol.

[B4] Webby RJ, Webster RG (2003). Are we ready for pandemic influenza?. Science.

[B5] Gani R, Hughes H, Fleming D, Griffin T, Medlock J, Leach S (2005). Potential impact of antiviral drug use during influenza pandemic. Emerg Infect Dis.

[B6] Longini IM, Halloran ME, Nizam A, Yang Y (2004). Containing pandemic influenza with antiviral agents. Am J Epidemiol.

[B7] Bell DM (2006). Non-pharmaceutical interventions for pandemic influenza, international measures. Emerg Infect Dis.

[B8] Bell DM (2006). Non-pharmaceutical interventions for pandemic influenza, national and community measures. Emerg Infect Dis.

[B9] Ferguson NM, Cummings DA, Cauchemez S, Fraser C, Riley S, Meeyai A, Iamsirithaworn S, Burke DS (2005). Strategies for containing an emerging influenza pandemic in Southeast Asia. Nature.

[B10] Ferguson NM, Cummings DA, Fraser C, Cajka JC, Cooley PC, Burke DS (2006). Strategies for mitigating an influenza pandemic. Nature.

[B11] Germann TC, Kadau K, Longini IM, Macken CA (2006). Mitigation strategies for pandemic influenza in the United States. Proc Natl Acad Sci U S A.

[B12] Longini IM, Nizam A, Xu S, Ungchusak K, Hanshaoworakul W, Cummings DA, Halloran ME (2005). Containing pandemic influenza at the source. Science.

[B13] Jefferson T, Demicheli V, Rivetti D, Jones M, Di Pietrantonj C, Rivetti A (2006). Antivirals for influenza in healthy adults: systematic review. Lancet.

[B14] Eichner M (2003). Case isolation and contact tracing can prevent the spread of smallpox. Am J Epidemiol.

[B15] Eichner M, Schwehm M, Duerr HP, Brockmann SO (2007). The influenza pandemic preparedness planning tool InfluSim. BMC Infect Dis.

[B16] Eichner M, Schwehm M InfluSim. http://www.influsim.info.

[B17] Kaiser L, Wat C, Mills T, Mahoney P, Ward P, Hayden F (2003). Impact of oseltamivir treatment on influenza-related lower respiratory tract complications and hospitalizations. Arch Intern Med.

[B18] Shortridge KF (2006). Influenza pandemic preparedness: gauging from EU plans. Lancet.

[B19] Frost WH (1919). The epidemiology of influenza. JAMA : the journal of the American Medical Association.

[B20] Collins SD (1930). The influenza epidemic of 1928-1929 with comparative data for 1918-1919. American journal of public health and the nation's health.

[B21] Mills CE, Robins JM, Lipsitch M (2004). Transmissibility of 1918 pandemic influenza. Nature.

[B22] WHO WHO Guidelines on the Use of Vaccines and Antivirals during Influenza Pandemics. http://www.who.int/vaccine_research/diseases/influenza/WHO_guidelines_on_the_use_of_vaccines_and_antivirals.pdf.

[B23] Hayden FG, Fritz R, Lobo MC, Alvord W, Strober W, Straus SE (1998). Local and systemic cytokine responses during experimental human influenza A virus infection. Relation to symptom formation and host defense. J Clin Invest.

[B24] Fraser C, Riley S, Anderson RM, Ferguson NM (2004). Factors that make an infectious disease outbreak controllable. Proc Natl Acad Sci U S A.

[B25] Arino J, Brauer F, van den Driessche P, Watmough J, Wu J (2006). Simple models for containment of a pandemic. Journal of the Royal Society Interface.

[B26] Wallinga J, Teunis P (2004). Different epidemic curves for severe acute respiratory syndrome reveal similar impacts of control measures. Am J Epidemiol.

[B27] Nishiura H, Kuratsuji T, Quy T, Phi NC, Van Ban V, Ha LE, Long HT, Yanai H, Keicho N, Kirikae T, Sasazuki T, Anderson RM (2005). Rapid awareness and transmission of severe acute respiratory syndrome in Hanoi French Hospital, Vietnam. Am J Trop Med Hyg.

[B28] Wu J, Xu F, Zhou W, Feikin DR, Lin CY, He X, Zhu Z, Liang W, Chin DP, Schuchat A (2004). Risk factors for SARS among persons without known contact with SARS patients, Beijing, China. Emerg Infect Dis.

[B29] Lau JT, Tsui H, Lau M, Yang X (2004). SARS transmission, risk factors, and prevention in Hong Kong. Emerg Infect Dis.

[B30] Pourbohloul B, Meyers LA, Skowronski DM, Krajden M, Patrick DM, Brunham RC (2005). Modeling control strategies of respiratory pathogens. Emerging Infectious Diseases.

[B31] PandemicPlan_US U.S. Department of Health & Human Services Pandemic Influenza Plan.. http://www.hhs.gov/pandemicflu/plan/.

